# Fast and accurate identification of cryptic and sympatric mayfly species of the *Baetis rhodani* group

**DOI:** 10.1186/s13104-017-3115-6

**Published:** 2018-01-08

**Authors:** Roberta Bisconti, Roberta Tenchini, Carlo Belfiore, Giuseppe Nascetti, Daniele Canestrelli

**Affiliations:** Dipartimento di Scienze Ecologiche e Biologiche, Università degli Studi della Tuscia, Viale dell’Università s.n.c., 01100 Viterbo, Italy

**Keywords:** High resolution melting (HRM) curve, Species identification, *Baetis rhodani*, Tyrrhenian islands, Real time PCR

## Abstract

**Objective:**

Species of the *Baetis rhodani* group are among the most widespread mayflies of the Palearctic region. However, frequent occurrence of morphologically cryptic species complicates the identification of sympatric species. Here, we proposed and tested a method for the fast, accurate, and cost-effective assignment of a large number of individuals to their putative species, based on high resolution melting profiles of a standard mitochondrial gene fragment. We tested this method using a system of three recently identified cryptic species inhabiting the Tyrrhenian Islands (western Mediterranean basin).

**Results:**

Highly species-specific high resolution melting profiles were obtained, allowing the unequivocal attribution of each individual to the respective species. This assay provides a convenient and easily customizable alternative to traditional barcoding approaches, provided that the mayfly taxa occurring within the geographic area of interest have been previously identified and their high resolution melting profiles assessed.

**Electronic supplementary material:**

The online version of this article (10.1186/s13104-017-3115-6) contains supplementary material, which is available to authorized users.

## Introduction

*Baetis rhodani* (Pictet 1843) is a complex of morphologically cryptic mayfly species widespread in the Palearctic region [1]. Despite a considerable research effort in recent years [2–5], current knowledge of the phylogenetic relationships within this group and the taxonomic status of several of its representatives remain incomplete, with new species emerging as new areas of the Palearctic are investigated.

Methods of species recognition and individual assignment in mayflies have been mostly based on two distinct approaches, each one with its own advantages and drawbacks. The first and more traditional approach is based on the analysis of morphological diagnostic traits, primarily of the larval stages [6, 7]. Although relatively inexpensive and time-saving, this approach relies entirely on an expert knowledge of the taxonomic group under study. Most importantly, with the advent of the second approach, molecular taxonomy, morphological approaches have soon appeared largely to underestimate the amount of species diversity within baetidae mayflies, including *B. rhodani* [2]. Indeed, under the single morphological species *B. rhodani*, several deeply divergent species have recently been recognized using molecular markers [2–5], and even more are likely to be described in the near future. On the other hand, molecular methods, such as those based on DNA barcodes, entail unprecedented processing costs per sample, and their extensive use for the preliminary phase of species delimitation has been largely debated (see [8, 9]).

Recently, three endemic species belonging to the *B. rhodani* species group have been identified within the Tyrrhenian Islands, based on a multi-locus analysis of genetic variation among 112 individuals from 28 populations [5]. However, to obtain a careful characterization of the respective distribution ranges, patterns of co-occurrence on single islands, relative abundance in sites of co-occurrence, as well as for all subsequent ecological and evolutionary investigations on these species, the characterization of a much larger sample of individuals and populations will be mandatory. Since nuclear and mitochondrial patterns of variation were largely congruent to one another in this group of species [5], the use of a DNA barcoding approach would be fully suitable.

We developed and validated a method for the assignment of a large number of individual mayflies to their putative species, based on the analysis of the high-resolution melting (HRM) curve of a standard molecular marker (NADH dehydrogenase subunit 1 gene). HRM is an effective, still not fully exploited, acquisition in the molecular taxonomy toolbox (see e.g. [10, 11]), which allows to assess the occurrence of sequence differences of diagnostic value between taxa, based on melting temperature profiles and the associated fluorescence peaks. Although based on a DNA barcoding approach, this method is faster, more cost-effective, and equally accurate when compared with traditional sequence-based approaches, provided that the pattern of variation within the focal taxa has already been characterized, as is the case for the *B. rhodani* group on the Tyrrhenian Islands [5].

## Main text

### Methods

**Fig. 1:**
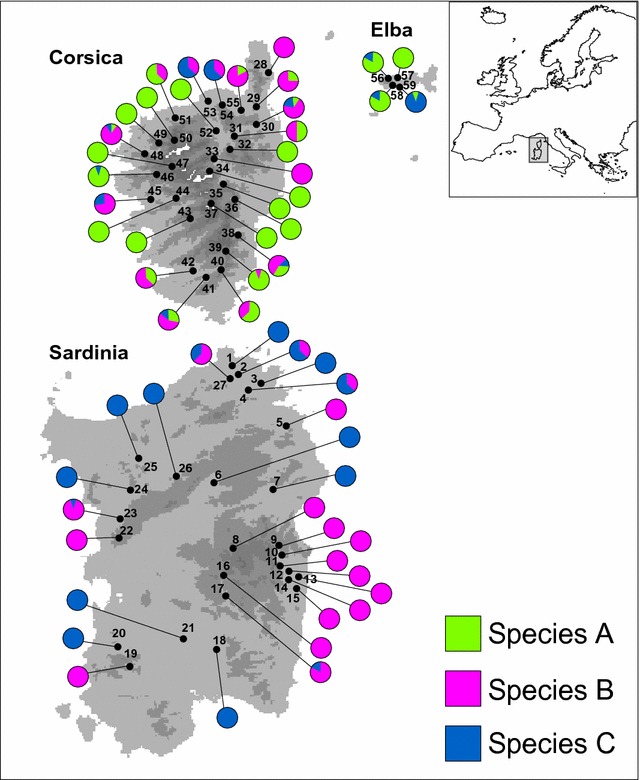
Geographic location of the 59 sites sampled within the Tyrrhenian Islands. Localities are numbered as in Table 1. Pie diagrams show the proportion of individuals belonging to the three species within each sampled locality. (The digital elevation model was downloaded by the WorldClim 1.4 database, freely available at www.worldclim.org)

In total, 399 larvae—100 individuals from a previous study [5] and 299 new samples-of the *Baetis rhodani* species group were collected from 59 localities (Table 1, Fig. 1) spanning the three main Tyrrhenian Islands (Sardinia, Corsica and Elba islands; no ethical approval is needed for sampling or processing these invertebrate species). The larvae were preserved in 95% ETOH prior to DNA extraction. A formal species description of the three species occurring in the study area is still pending. For consistency with the previous work which identified them as distinct biological species, we will refer to them here as Species A, Species B, and Species C. An unequivocal species assignment was made possible by the deep mitochondrial sequence divergence observed between the three species. Indeed, the percent sequence divergence between species (*p*-*distance*) was as follows [5]: A vs B = 14%, A vs C = 29%, and B vs C = 26%.

**Table 1:** Geographic location and sample size (n) of the 59 sites sampled within the Tyrrhenian Islands

Island	Latitude N	Longitude E	n
Sardinia			
1	41°6.611′	9°13.731′	1
2	41°5.569′	9°14.757′	3
3	41°3.825′	9°24.300′	4
4	41°2.900′	9°19.756′	6
5	40°46.235′	9°32.380′	4
6	40°28.613′	9°7.766′	2
7	40°22.751′	9°26.065′	10
8	40°2.221′	9°15.438′	6
9	40°2.752′	9°31.012′	10
10	40°1.237′	9°31.922′	5
11	39°57.661′	9°32.632′	2
12	39°57.625′	9°33.680′	4
13	39°57.097′	9°36.282′	2
14	39°56.477′	9°34.785′	10
15	39°55.590′	9°38.244′	4
16	39°53.738′	9°11.561′	10
17	39°49.494′	9° 12.106′	10
18	39°30.284′	9°8.095′	8
19	39°23.525′	8°40.131′	6
20	39°30.210′	8°37.196′	2
21	39°33.355′	8°59.529′	7
22	40° 8.863′	8°32.295′	7
23	40°14.036′	8°35.382′	9
24	40°24.421′	8°37.614′	8
25	40°35.799′	8°38.053′	9
26	40°31.236′	8°52.058′	10
27	41°5.433′	9°14.119′	6
Corsica			
28	42°48.760′	9°28.427′	12
29	42°35.515′	9° 21.799′	4
30	42°30.267′	9° 22.517′	9
31	42°26.069′	9°13.433′	4
32	42° 18.291′	9°8.779′	9
33	42° 16.504′	9°6.438′	5
34	42°11.860′	9°7.299′	3
35	42°6.869′	9°11.006′	2
36	42°6.153′	9°14.679′	8
37	42°0.990′	9°2.884′	5
38	41°49.292′	9°15.599′	7
39	41°46.110′	9°10.407′	10
40	41°41.447′	9°9.113′	10
41	41°37.541′	9°4.962′	7
42	41°39.831′	9° 0.889′	8
43	41°57.634′	9°0.340′	4
44	42°3.578′	8°57.781′	8
45	42°2.975′	8°47.106′	7
46	42°10.170′	8°49.200′	9
47	42°14.317′	8°50.838′	9
48	42°17.714′	8°42.141′	10
49	42°22.993′	8°41.940′	4
50	42°21.865′	8°48.113′	6
51	42°29.176′	8°48.176′	8
52	42°28.076′	9°6.385′	8
53	42°37.292′	8°59.813′	8
54	42°36.138′	9°8.313′	8
55	42°34.182′	9°18.256′	11
Elba			
56	42°47.379′	10°7.370′	5
57	42°47.064′	10°9.962′	10
58	42°44.556′	10°10.353′	6
59	42°44.298′	10°10.604′	10

Total genomic DNA extraction was carried out from the thorax, legs, or entire larval body by proteinase K (Promega) digestion and a standard phenol–chloroform protocol [12]. The amount of genomic DNA of each sample was assessed with the Qubit 2.0 fluorometer (Invitrogen, Carlsbad, CA, USA), using the Qubit dsDNA BR Assay kit (Thermo Fisher Scientific Inc.). Each sample was then concentrated to 10 ng/μL for downstream analyses.

Real-time quantitative PCR assays were carried out in 96-well plates through amplification of the mitochondrial NADH dehydrogenase subunit 1 gene (ND1) fragment with the following primers: ND1F (TAAAGTTAGCAGGTTCATACCC) and ND1R (CACCTATATTTGTACTTTGAAGG). Amplifications were run in a 15-μl reaction volume containing MgCl_2_ (2 mM, Promega), four dNTPs (0.2 mM each, Promega), two primers (0.2 μM each, Sigma-Aldrich), the enzyme Hotstart *Taq* polymerase (0.5 U, Promega), the reaction buffer (1×, Promega), EvaGreen^®^ dye (1×, Biotium), and ~ 10–30 ng of DNA template.

Real time PCRs were run using a Roche LightCycler^®^ LC480. The PCR cycling conditions were as follows: an initial step of 5 min at 95 °C, 35 cycles of 1 min at 94 °C, 45 s at 57 °C, and 90 s at 72 °C, and an extension step of 10 min at 72 °C. A final melting cycle was performed for 3 min at 95 °C and a melt from 40 to 95 °C collecting fluorescence continuously at a ramping rate of 0.1 °C per second. The HRM peaks obtained from single individuals were examined after each real-time PCR using the LightCycler^®^ 480 Software (release version 1.5.0).

In order to characterize the HRM curve profiles of each species, we included in the analyses all the individuals belonging to the three species analysed in the previous study (n = 100; [5]), where the diagnostic value of sequence variation among the three species was assessed. To further test the accuracy of the proposed method, we randomly selected and sequenced 24 individuals from the pool of newly screened samples (Genebank Accession Numbers: MG581893–MG581916). We then compared their species assignment based on HRM profiles to those achieved through standard sequencing.

In order to evaluate the diagnostic ability of the HRM curve method for the three species studied, we performed a principal component analysis (PCA) using the IBM SPSS v. 23 (IBM SPSS Statistics for Windows, Armonk, NY: IBM Corp), based on peak temperature and fluorescence values of the 100 individuals from the previous paper [5]. The first principal component was then used to perform a receiver operating characteristic (ROC) curve analysis, whose accuracy was evaluated by means of the area under the curve (AUC) values.

### Results and discussion

**Fig. 2:**
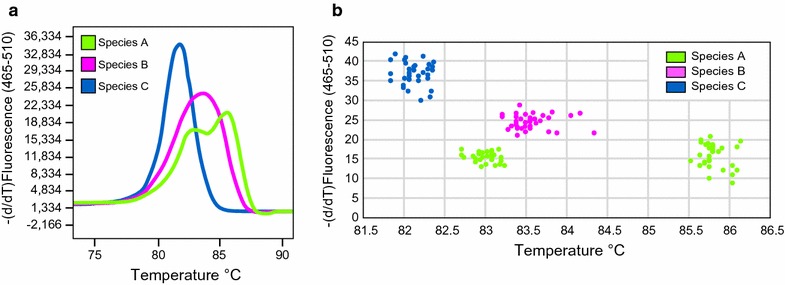
High resolution melting curve analysis. **a** A negative first-derivative plot of fluorescence over temperature for three representative individuals of Species A, B, and C (as defined by [5]); redrawn from LightCycler 480 Software. **b** A 2D scatterplot of melting temperatures at fluorescence peaks for the 100 individuals used to assess the diagnostic value of melting curves for each species

The derived HRM curves obtained for the individuals already sequenced from a previous study clearly showed three distinct profiles (see Fig. 2a). A comparison of these profiles with the standard sequence-based species identification unequivocally indicated the diagnostic value of the HRM curves. This facilitated assigning each unique profile to one of the three species, without misidentifications.

The three HRM curves observed within the negative first-derivative plot were easily distinguishable from one another, based on curve shape and melting peak values (Fig. 2a). The curve identifying Species A showed two peaks, the first at 83 °C with a maximum value of 17.3 and a minimum value of 12.8 (−dF/dT), and the second peak at 86 °C with a maximum value of 20.5 and a minimum value of 8.6 (−dF/dT). The HRM curve for Species B was diagnosable by a single peak at 83–84 °C with a maximum value of 28.4 and a minimum value of 19.7 (−dF/dT). Finally, the HRM profile for Species C showed both the highest and narrowest curve profile, with a melting peak at 82 °C and a maximum value of 41.6 and a minimum value of 29.6 (−dF/dT). As shown by the scatterplot in Fig. 2b (see also Additional file 1), inter-individual variation within each species did not affect the diagnostic significance of the individual peak values.

The analysis of the 399 samples collected throughout the Tyrrhenian Islands (Sardinia, Corsica and Elba islands) did not reveal any new patterns for the HRM curve profiles in addition to the three described above. We assigned all analysed samples to a putative species: 128 to Species A, 170 to Species B, and 101 to Species C. As expected, based on the absence of new HRM curve variants among the newly studied samples, sequence-based assignment of all 24 individuals used as the control perfectly matched species identity as defined by melting profiles. Moreover, the ROC curve analysis based on the first principal component (variance explained: 82%) strongly supported the diagnostic ability of HRM method used, showing extremely high values of the AUC, in all pairwise comparisons between species (range of the AUC values: 0.97–1.00).

Our results clearly demonstrated that the HRM-based method proposed here to assess species identity for mayflies of the *B. rhodani* group on the Tyrrhenian Islands, proved as accurate and reliable as the standard sequence-based approach, besides allowing to avoid sequencing costs and efforts.

We utilized this method for a fast and accurate evaluation of the actual geographic distribution of the three cryptic species, which is fundamental information for any future study employing these species as target organisms. The results of this evaluation revealed distinct distribution patterns for the three studied species (Fig. 1), allowing us to incorporate them within a long-term study on the evolution of the insular biota (see also [13–19], to investigate species-specific eco-evolutionary dynamics, and to improve ongoing bio-monitoring programs in the area [20].

## Limitations

- The method does not provide information about genetic diversity, population structure, or phylogenetic relationships of the study organisms.
- The method is only applicable when background information on the interspecific genetic variation is available for diagnostic markers.

## Additional file

**Additional file 1.** An excel file format containing data used to draw the scatterplot of Fig. 2b.

## Abbreviations

HRM: high-resolution-melting
ND1: NADH dehydrogenase subunit 1 gene fragment

## References

[1] Brittain JE (1982). Biology of mayflies. Annu Rev Entomol.

[2] Williams HC, Ormerod SJ, Bruford MW (2006). Molecular systematics and phylogeography of the cryptic species complex *Baetis rhodani* (Ephemeroptera, Baetidae). Mol Phylogenet Evol.

[3] Lucentini L, Rebora M, Puletti ME, Gigliarelli L, Fontaneto D, Gaino E (2011). Geographical and seasonal evidence of cryptic diversity in the *Baetis rhodani* complex (Ephemeroptera, Baetidae) revealed by means of DNA taxonomy. Hydrobiologia.

[4] Rutschmann S, Gattolliat JL, Hughes SJ, Baez M, Sartori M, Monaghan MT (2014). Evolution and island endemism of morphologically cryptic *Baetis* and *Cloeon* species (Ephemeroptera, Baetidae) on the Canary Islands and Madeira. Freshw Biol.

[5] Bisconti R, Canestrelli D, Tenchini R, Belfiore C, Buffagni A, Nascetti G (2016). Cryptic diversity and multiple origins of the widespread mayfly species group *Baetis rhodani* (Ephemeroptera: Baetidae) on northwestern Mediterranean islands. Ecol Evol.

[6] Müller-Liebenau I (1969). Revision der europäischen Arten Gattung Baetis Leach, 1815 (Insecta, Ephemeroptera). Gewässer und Abwässer.

[7] Gattolliat JL, Sartori M (2008). What is *Baetis rhodani* (Pictet 1843)(Insecta, Ephemeroptera, Baetidae)? Designation of a neotype and redescription of the species from its original area. Zootaxa.

[8] Song H, Buhay JE, Whiting MF, Crandall KA (2008). Many species in one: DNA barcoding overestimates the number of species when nuclear mitochondrial pseudogenes are coamplified. Proc Natl Acad Sci.

[9] Hudson RR, Coyne JA (2002). Mathematical consequences of the genealogical species concept. Evolution.

[10] Gopaul KK, Sells J, Lee R, Beckstrom-Sternberg SM, Foster JT, Whatmore AM (2014). Development and assessment of multiplex high resolution melting assay as a tool for rapid single-tube identification of five *Brucella* species. BMC Res Notes.

[11] Madesis P, Ganopoulos I, Anagnostis A, Tsaftaris A (2012). The application of Bar-HRM (Barcode DNA-High Resolution Melting) analysis for authenticity testing and quantitative detection of bean crops (Leguminosae) without prior DNA purification. Food Control.

[12] Sambrook J, Fritsch EF, Maniatis T (1989). Molecular cloning: a laboratory manual.

[13] Salvi D, Schembri PJ, Sciberras A, Harris DJ (2014). Evolutionary history of the Maltese wall lizard *Podarcis filfolensis*: insights on the ‘Expansion-Contraction’ model of Pleistocene biogeography. Mol Ecol.

[14] Salvi D, Bisconti R, Canestrelli D (2016). High phylogeographical complexity within Mediterranean islands: insights from the Corsican fire salamander. J Biogeogr.

[15] Bisconti R, Canestrelli D, Nascetti G (2013). Has living on islands been so simple? Insights from the insular endemic frog *Discoglossus montalentii*. PLoS ONE.

[16] Bisconti R, Canestrelli D, Salvi D, Nascetti G (2013). A geographic mosaic of evolutionary lineages within the insular endemic newt *Euproctus montanus*. Mol Ecol.

[17] Bauzà-Ribot MM, Jaume D, Fornos JJ, Juan C, Pons J (2011). Islands beneath islands: phylogeography of a groundwater amphipod crustacean in the Balearic archipelago. BMC Evol Biol.

[18] Bisconti R, Canestrelli D, Nascetti G (2011). Multiple lines of evidence for demographic and range expansion of a temperate species *Hyla sarda* during the last glaciation. Mol Ecol.

[19] Bisconti R, Canestrelli D, Nascetti G (2011). Genetic diversity and evolutionary history of the Tyrrhenian treefrog Hyla sarda (Anura: Hylidae): adding pieces to the puzzle of Corsica-Sardinia biota. Biol J Linn Soc.

[20] Buffagni A, Tenchini R, Cazzola M, Erba S, Balestrini R, Belfiore C (2016). Detecting the impact of bank and channel modification on invertebrate communities in Mediterranean temporary streams (Sardinia, SW Italy). Sci Total Environ.

